# The Role of Climate Variability in the Spread of Malaria in Bangladeshi Highlands

**DOI:** 10.1371/journal.pone.0014341

**Published:** 2010-12-16

**Authors:** Ubydul Haque, Masahiro Hashizume, Gregory E. Glass, Ashraf M. Dewan, Hans J. Overgaard, Taro Yamamoto

**Affiliations:** 1 Department of International Health, Institute of Tropical Medicine (NEKKEN) and The Global Center of Excellence Program, Nagasaki University, Nagasaki, Japan; 2 Department of Molecular Microbiology and Immunology, Johns Hopkins Bloomberg School of Public Health, Baltimore, Maryland, United States of America; 3 Department of Geography and Environment, University of Dhaka, Dhaka, Bangladesh; 4 Department of Spatial Sciences, Curtin University of Technology, Perth, Australia; 5 Department of Mathematical Sciences and Technology, Norwegian University of Life Sciences, Aas, Norway; Universidade Federal de Minas Gerais, Brazil

## Abstract

**Background:**

Malaria is a major public health problem in Bangladesh, frequently occurring as epidemics since the 1990s. Many factors affect increases in malaria cases, including changes in land use, drug resistance, malaria control programs, socioeconomic issues, and climatic factors. No study has examined the relationship between malaria epidemics and climatic factors in Bangladesh. Here, we investigate the relationship between climatic parameters [rainfall, temperature, humidity, sea surface temperature (SST), El Niño-Southern Oscillation (ENSO), the normalized difference vegetation index (NDVI)], and malaria cases over the last 20 years in the malaria endemic district of Chittagong Hill Tracts (CHT).

**Methods and Principal Findings:**

Monthly malaria case data from January 1989 to December 2008, monthly rainfall, temperature, humidity sea surface temperature in the Bay of Bengal and ENSO index at the Niño Region 3 (NIÑO3) were used. A generalized linear negative binomial regression model was developed using the number of monthly malaria cases and each of the climatic parameters. After adjusting for potential mutual confounding between climatic factors there was no evidence for any association between the number of malaria cases and temperature, rainfall and humidity. Only a low NDVI was associated with an increase in the number of malaria cases. There was no evidence of an association between malaria cases and SST in the Bay of Bengal and NIÑO3.

**Conclusion and Significance:**

It seems counterintuitive that a low NDVI, an indicator of low vegetation greenness, is associated with increases in malaria cases, since the primary vectors in Bangladesh, such as *An. dirus*, are associated with forests. This relationship can be explained by the drying up of rivers and streams creating suitable breeding sites for the vector fauna. Bangladesh has very high vector species diversity and vectors suited to these habitats may be responsible for the observed results.

## Introduction

Malaria is the most important tropical and parasitic disease in the world. In 2008, there were an estimated 243 million cases of malaria, the vast majority of cases (85%) occurring in the African Region. In 2008, malaria accounted for an estimated 863,000 deaths [1]. Bangladesh is one of ten Asian countries where malaria is endemic [2]. In 2008, malaria morbidity and mortality in Bangladesh were recorded as 84,690 and 154, respectively [3].

**Figure 1:**
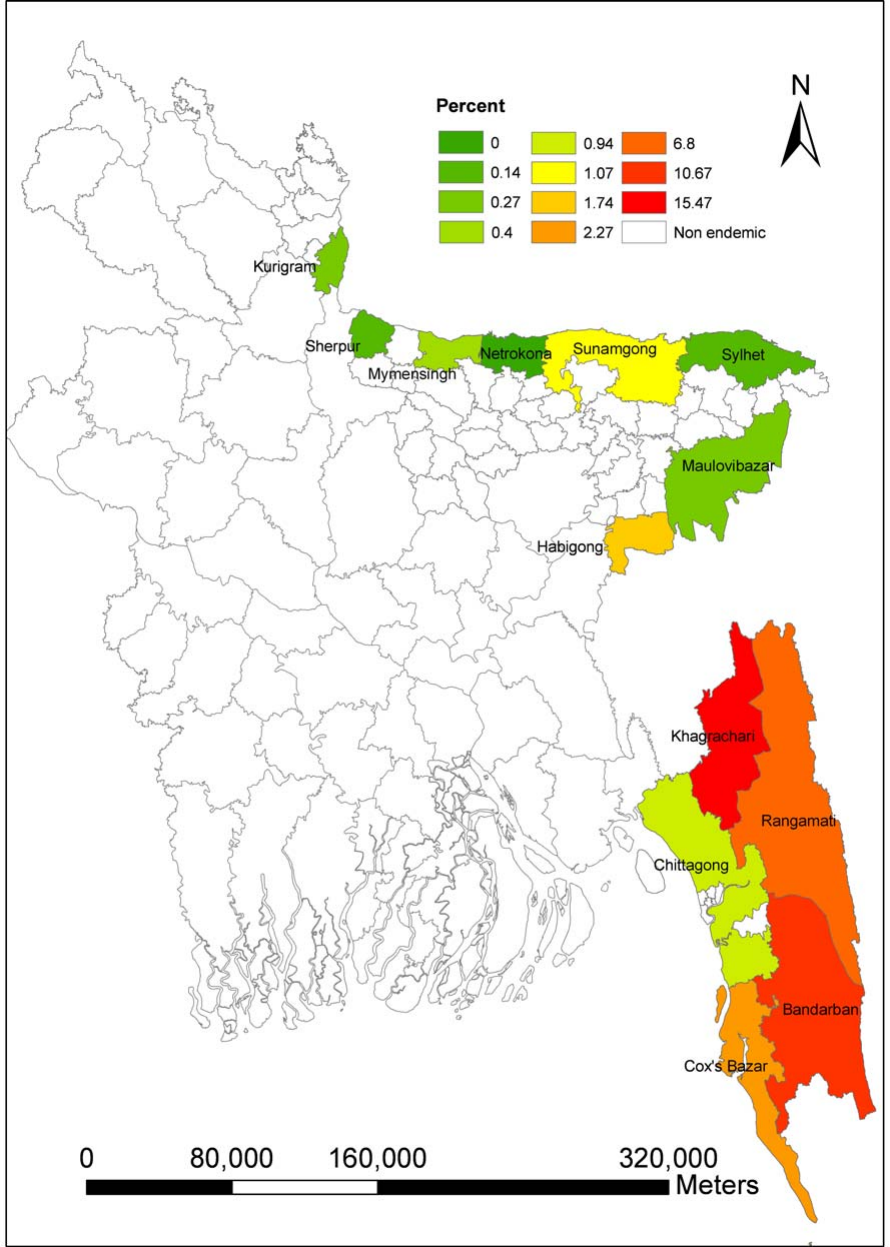
Spatial distribution of malaria prevalence in Bangladesh.

Malaria is endemic in 13 northern and eastern districts of Bangladesh along the border with India and Myanmar, with 90% of morbidity and mortality reported from three hill districts (Rangamati, Bandarban and Khagrachari) (Figure 1). The malaria prevalence rate in Bangladesh was 3.97% in 2007 [2]. The majority of infections was *P. falciparum* (90.2%), with *P. vivax* and mixed infections making up 5.3% and 4.5% respectively [2].

The malaria vector situation in Bangladesh is complex due to high species diversity and the presence of species complexes with many sibling species displaying different ecological behaviors [4]. In Bangladesh *An. minimus* s.l., *An. dirus*, *An. philippinensis*, and *An. sundaicus* are considered primary malaria vectors and *An. aconitus*, *An. annularis*, and *An. vagus* as secondary vectors [5]. However, recent studies have incriminated a range of other species, such as *Anopheles nigerrimus*, *An. subpictus*, *An. barbirostris*, and *An. maculatus* [6], [7]. The main vectors in the study area are *An. baimai (dirus)*, *An. philippinensis*, *An. vagus*, and *An. minimus* [7].

In Bangladesh, malaria became epidemic during the 1990s possibly due to the ban of DDT (dichlorodiphenyltrichloroethane) in 1985, lack of malaria control efforts, insecticide resistance and resistance to chloroquine (1st line drug at that time) [8], [9] and many factors may account for the pattern of malaria infection in the country. Land use change is a part of environmental change and directly affects vector habitats. With land use change, malaria may either increase or decrease [10]. Drug resistance is also a key factor and responsible for sharp increases in malaria [8]. An efficient malaria control program can also significantly reduce malaria transmission [11]. It is well known that malaria is a poverty related disease and strongly associated with socioeconomic status [12]. However, trends in climatic factors are also driving forces which affect malaria transmission [13]. No previous studies have elucidated the relationship between changes in the incidence of malaria and fluctuations of climate variables in Bangladesh.

Studies throughout the world have linked changes in malaria incidence with patterns of rainfall, temperature and humidity [14], [15]. Rainfall is considered to be a major factor influencing malaria cases in Africa [16] and a causal relationship between rainfall and malaria transmission is well recognized [17], [18], [19]. In Sri Lanka, malaria cases were strongly correlated with rainfall with a time lag of 0–3 months [14]. Malaria cases increased by 1.4% to 10.7% per month for each 10 millimeter increase in monthly rainfall (with a 2–3 month lag) in the highlands of West Kenya [20]. Natural climatic disasters such as floods and cyclones may also have significant relationship with malaria outbreaks [21].

Temperatures between 15 to 40°C and humidity between 55 to 80% are suitable for the completion of the *P. falciparum* and *P. vivax* malaria parasite life cycles [22]. Such conditions are found throughout the seasons in India, where a close association between temperature, rainfall conditions, and malaria has been reported [22]. The minimum temperature was strongly associated with the occurrence of malaria cases in Rwanda [23]. Another study in east African highlands have shown that a 1°C increase in minimum temperature with a lag time of 1–2 months and a 1°C increase in maximum temperatures with a lag time of 2–5 months led to an 8–95% increase in the number of malaria outpatients [24].

Satellites from the U.S. National Oceanic and Atmospheric Administration (NOAA) environmental satellites provide a vegetation survey at the climatic scale. Normalized Difference Vegetation Index (NDVI) is a measure of vegetation conditions. NDVI values vary between +1.00 and −1.00; the higher the NDVI value, the denser or healthier the green vegetation. Strong relationship between vegetation health (VH) (another measure of vegetation conditions and similar to NDVI), and malaria cases has been demonstrated in Bangladesh, indicating that VH can be used as an indicator of climatic and environmental conditions [25].

El Niño and La Niña years coincide with low and high rainfall years in southern Asia. El Niño Southern Oscillation (ENSO) has been used as predictor of climatic events and a significant correlation has been reported between sea surface temperature (SST) and malaria cases [15], [17], [18], [22]. El Niño Southern Oscillation showed a significant association with malaria case numbers. A 1°C increase in Niño 3.4 (region in Pacific) SST was associated with about a 20% increase in malaria cases in Colombia [26]. An analysis of 37 years of national statistics in India showed, in general, that if the number of malaria cases in a particular year was less than the decadal average, that particular year was influenced by La Niña; and when the number of malaria cases in a particular year exceeded the decadal average, that particular year was influenced by El Niño [22].

There is a strong interest in investigating the relationship between climate variability and malaria transmission and with concerns about potential climate change, this interest has increased. A rise of about 6% in malaria cases during 2000 in middle income countries was attributed to climate change [21]. In this study, we investigate the relationship between climate variability and malaria cases in the endemic area of Bangladesh.

## Results

**Figure 2:**
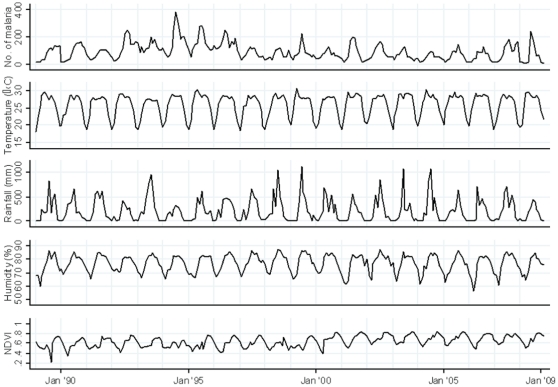
Time series of the number of all malaria cases per month and meteorological data in Rangamati, 1989–2008.

The time series of the number of malaria cases, rainfall, temperature, humidity and NDVI from January 1989 to December 2008 are shown in Figure 2. The time series of SST of Bay of Bengal, NINO3 during the study period is shown (Figure S1). There was a distinct seasonality in the number of malaria cases with a peak during June to August. High temperatures occurred in April to September in each year. Except for some small fluctuations, rainfall occurred between May and October. In some years, high NDVI was observed in all seasons but the peak occurred between in October and November. SST was lowest in January and February, started to increase in March, remained high until October and decreased from November onwards.

**Figure 3:**
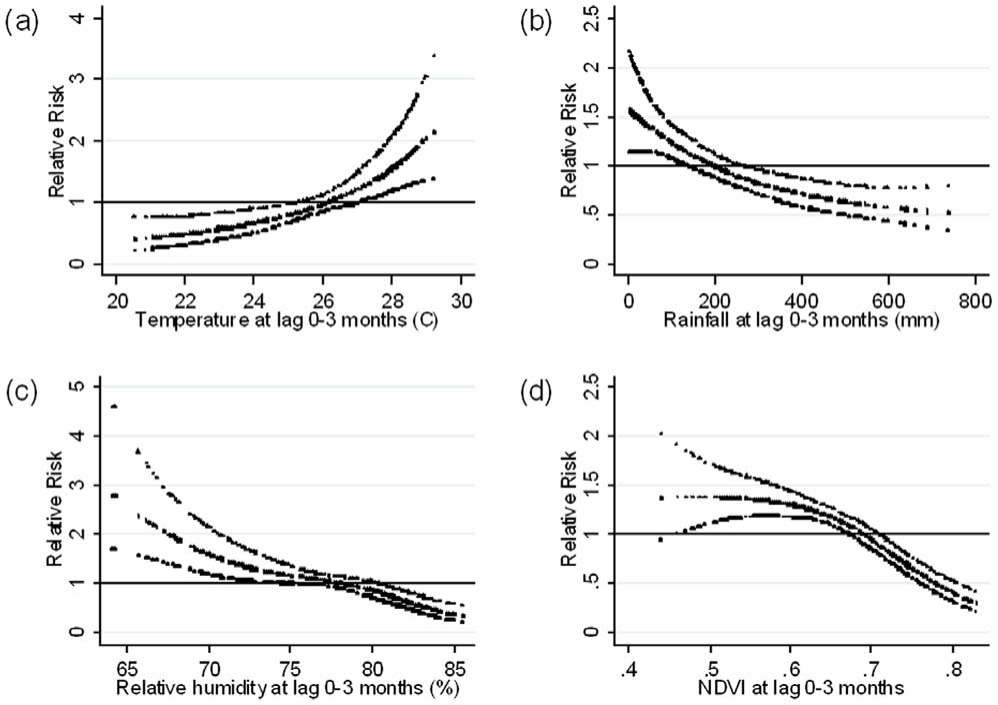
Relationship between the number of malaria cases per month and (a) average mean temperature, (b) total rainfall, (c) average relative humidity and (d) Normalized difference vegetation index (NDVI) over lags of 0–3 months (shown as a 3 d.f. natural cubic spline) adjusted for seasonal variation and between-year variations. RR represents the relative risk of malaria (scaled against the mean monthly number of cases). The centre line in each graph shows the estimated spline curve, and the upper and lower lines represent the 95% confidence limits.

The number of malaria cases increased significantly with increased temperature with a lag time of 0–3 months (p = 0.007) (Figure 3a) but decreased significantly with higher rainfall with a lag of 0–3 months (p = 0.002) (Figure 3b). The number of malaria cases also decreased as humidity increased with a lag time of 0–3 months (p<0.001) (Figure 3c). The number of malaria was significantly negatively associated with NDVI at a lag of 0–3 months (p<0.001) (Figure 3d).

**Figure 4:**
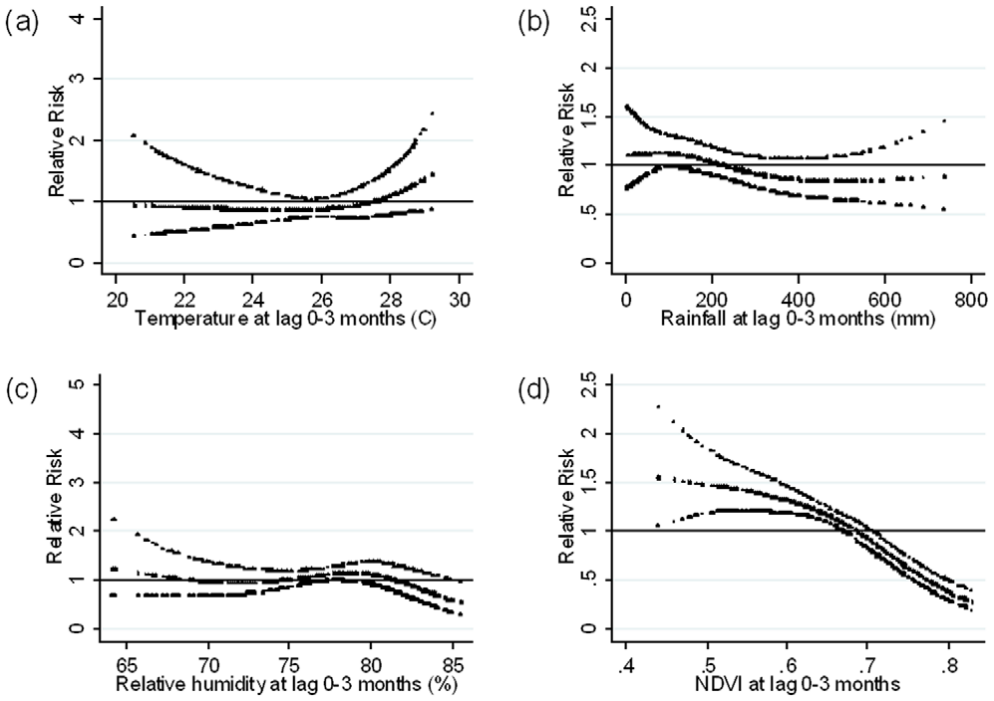
Relationship between the number of malaria cases per month and (a) average mean temperature, (b) total rainfall, (c) average relative humidity and (d) Normalized difference vegetation index (NDVI) over lags of 0–3 months (shown as a 3 d.f. natural cubic spline) adjusted for potential mutual confounding between these 4 variables, seasonal variation and between-year variations. RR represents the relative risk of malaria (scaled against the mean weekly number of cases). The centre line in each graph shows the estimated spline curve, and the upper and lower lines represent the 95% confidence limits.

The risk response relationships adjusted for potential mutual confounding between malaria cases and temperature, rainfall, and humidity with lag times of 0–3 months showed no significant associations (Figure 4a–c). However, malaria cases remained significantly associated with lower NDVI with a lag time of 0–3 months (Figure 4d). Each 0.1 increase in monthly NDVI was associated with a 30.4% decrease in malaria cases (95% CI: 19.2–40.1).

**Figure 5:**
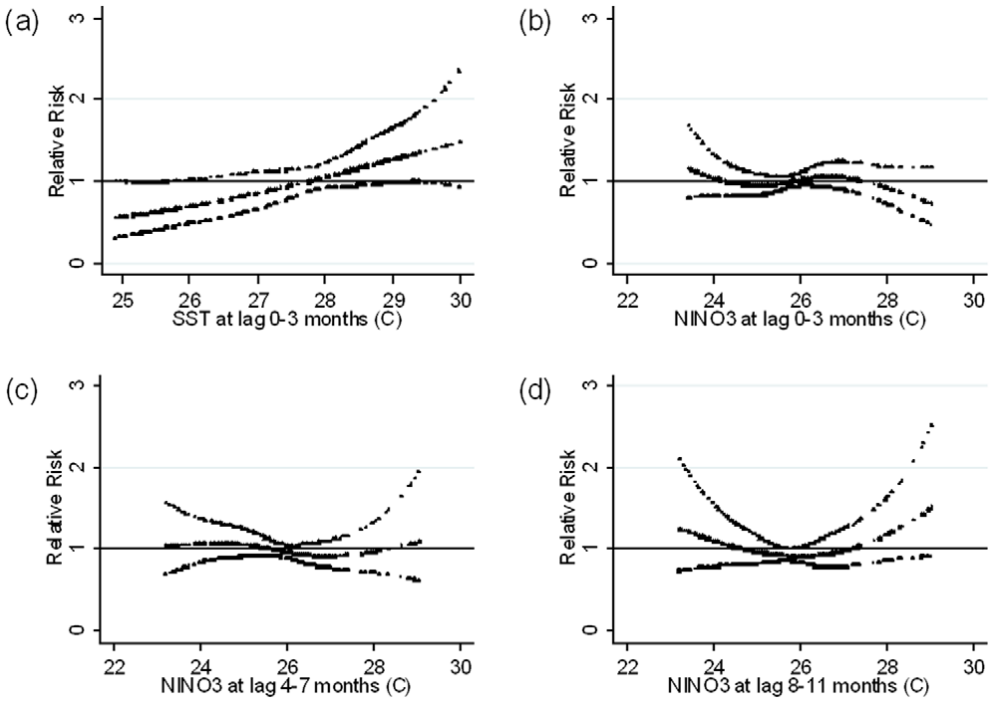
Relationship between the number of malaria cases per month and (a) average sea surface temperature (SST) of the Bay of Bengal over lags of 0–3 months, (b) average NINO3 over lags of 0–3 months, (c) 4–7 months and (d) 8–11 months (shown as a 3 d.f. natural cubic spline) adjusted for potential mutual confounding between the lags of NINO3, seasonal variation and between-year variations. RR represents the relative risk of malaria (scaled against the mean monthly number of cases). The centre line in each graph shows the estimated spline curve, and the upper and lower lines represent the 95% confidence limits.

There were no significant associations between malaria cases and SST using lag times of 0–3 months (Figure 5a) and NINO3 with different lag periods (Figure 5b–d).

The incorporation of the Fourier terms (up to fifth harmonics adding 1 harmonic at a time) into the fully adjusted model, in place of indicator variables for months, had broadly little effect on the estimates of the effects of NDVI: model with no seasonal control (24.8% (95%CI: 14.3–34.0) decrease). Although there was no evidence for the effect of temperatures in the model adjusted for season with indicator variables of months, there was a significant positive effect of temperatures when there was no seasonal control and with the Fourier terms of 1 harmonic (results not shown).

## Discussion

After adjusting for potential confounders, our study suggests that the best leading indicator of the number of malaria cases was NDVI at a lag of 0–3 months, and that NDVI was negatively associated with malaria cases. We did not find significant relationships like other studies; however, this may be due to the fact that other studies used different methodologies in different regions of the world where the malaria ecology/epidemiology is quite different. This study draws attention again to the complex nature of the relationship between malaria and climate. However, it has also illustrated the potential value of such studies, both for identifying local factors which may predict the epidemiology of the disease as well as for providing a deeper understanding of the biology of the parasite system.

Temperature and number of malaria cases were positively associated when not considering the effect of other climatic confounding factors. However, after adjusting for all other parameters, no association was observed. Undoubtedly, temperature is a key factor in malaria transmission [24], [27], [28], [29], [30], [31], directly affecting mosquito development, survival, reproduction, activity, and the extrinsic incubation rate. For example, minimum temperatures during the cool months or the previous month have been associated with malaria transmission in China and Burundi, respectively [29], [32]. The reason we were not able to detect a significant temperature-malaria relationship could be because of the crude average temperature data used here concealing shorter term effects impacting vector populations. Furthermore, statistical significance alone does not always address the complex biological dynamics of mosquito development and temperature. However, temperature ranges in this region of Bangladesh are always favorable for mosquito development. Further research should consider multiple study areas, including biological models of mosquito development [e.g. 36] to improve detection of temperature effects on malaria transmission specific for Bangladesh.

In the unadjusted analysis, rainfall showed a clear negative correlation with the number of malaria cases, but incorporating other climatic factors eliminated the significant relationship. It is difficult to explain why the malaria-rainfall associations became non-significant when other climatic factors were included in the model. These results differ from studies carried out in Thailand where malaria cases were positively associated with rainfall [33]. Rainfall is also a major contributing factor for the increase of malaria cases in other areas [17], [18], [19], [24]. However, rainfall was negatively correlated with malaria cases in India [22]. Long term data from Sri Lankan (January 1972 to December 2005) showed that the region with the highest rainfall had the least malaria, and that malaria cases increased with lower rainfall [14]. Moderate correlation (r = 0.48, p = 0.069) with annual rainfall with malaria incidence confirmed from Indian Rajasthan. The incidence of *P. falciparum* malaria showed a significant correlation (r = 0.61, p = 0.016) with rainfall [34]. At the same time no clear relationship was observed between rainfall and malaria incidence in Madhya Pradesh, central India [35]. The interpretation was that drought caused pools in the river bed, creating suitable conditions for mosquito breeding. A similar explanation may be true in Rangamati, where the main malaria vectors have a comparable ecology to those in Sri Lanka [36]. For example, *An. minimus* s.l. is associated with slow-moving streams [37] as is the primary vector in Sri Lanka, *An. culifacies* which breeds in river bed pools [36]. *An. dirus*, on the other hand, breeds in small temporal pools in heavily shaded forests and seems to be positively associated with rainfall [38]. *Anopheles minimus* and *An. dirus* are also primary vectors in Thailand and it is thus difficult to explain the contradictory results obtained here [33]. An important point to consider here is that we might be witness of a potential shift in vector importance, which again stresses the need for continuous monitoring of vector transmission dynamics and detailed studies of vector bionomics in the region.

Other reasons for the observed result may be that high and intensive rainfall flush out breeding sites. It may also be that people are more aware of the risk of malaria during high rainfall and so take preventative measures. During the post monsoon, when rainfall decreases or stops, malaria cases increase. Better entomological data would greatly increase our understanding of the influence of rainfall on the biology of malaria in Bangladesh. Unfortunately, such entomological data are not yet available in Bangladesh.

In our study, a low relative humidity was associated with an increase in malaria cases in the bivariate analysis but after adjusting for other local climatic parameters, no significant relationship was observed. This could be explained by the fact that humidity is directly dependent on temperature and rainfall, thus confounding the results. Generally, increased humidity is believed to favor vector survival [39]. Little has been published on the relationship between humidity and risk of malaria [30], but Bhattacharya and colleagues reported humidity levels between 55 and 80% were suitable for both *P. falciparum and P. vivax* [22]. Humidity was found to be related with the number of malaria cases in China, where a relative humidity below 60% shortened the life span of the mosquito so that below 60%, there was a decline in the risk of clinical malaria while above 60% relative humidity the infection rate increased significantly [29], [30]. It was also confirmed that the malaria risk at 80% humidity was twice as high as that of 60% [29], [30]. Further studies are needed to elucidate the relationship between humidity and malaria epidemiology in Bangladesh.

Normalized Difference Vegetation Index, both unadjusted and adjusted for other variables, were negatively associated with number of malaria cases. In Eritrea, NDVI and malaria cases were significantly (positively) associated with each other [40]. Our results differ from those of Bruce et al [41], who showed no association with NDVI and infection rates in Malawi. In another study in Indochina Peninsula, overlaying maps of the vegetation index with indices of *P. falciparum and P. vivax* infection showed that areas with NDVI values higher than 0.3 or 0.4 coincided with areas of high malaria incidence [42]. A similar result was found in Mali where an NDVI between 0.35 and 0.4 was associated with an increase in malaria cases [43].

Satellite-based vegetation health (VH) indices have also been compared with malaria epidemiology to study whether they could be used as a proxy for monitoring malaria epidemics in sixty four districts of Bangladesh. During drought years, when vegetation was under stress, fewer people had malaria [25]. The fact that our findings are not consistent with results from these areas could be due to within-country variations, such as different ecological habits of vector species and their siblings, changes in vector transmission dynamics, geographical and socio-economic settings, drug resistance, immunity among people, or control efforts. It can also be due to differences among climatic parameters between African countries and Bangladesh. Summer-winter seasonality may have some affect on the activity of mosquito vectors. It is also mostly due to the climatic dependency of vector behavior and different areas are likely to experience different effects in the rate of malaria vector growth because of climatic parameters including NDVI. Interestingly, NDVI has already been shown to be a reliable estimate of vector population and vector species distribution [44]. A relationship between weekly and monthly NDVI and mosquito abundance has also been demonstrated [45].

There was no relationship between SST, NIÑO3 and malaria cases. In contrast, analyses of the trends of SST over the eastern equatorial Pacific indicated that SST during March, April and May were negatively correlated with malaria cases in India from 1980 to 2000 [22]. Positive relationships were observed between Southern Oscillation Index and monthly incidences of malaria in China [46]. Historical epidemic malaria in Punjab between 1868 and 1943 correlates significantly with the sea surface temperature anomalies in the Eastern Equatorial Pacific. At the same time, 9 out of 16 malaria epidemics in the south west part of Sri Lanka were recorded between 1870 and 1945 during El Nino years [47].

In the East African highlands, ENSO events may trigger heavy rainfall and raised temperatures and were associated with increased malaria in the southwestern highlands of Uganda [48]. The same ENSO and heavy rainfall reduced malaria in the Tanzanian highlands [48]. The relationship between SST and rainfall in Bangladesh has not been extensively investigated. During the ENSO years of this study, rainfall decreased significantly in Bangladesh [49], similar to an earlier study using a 43-year data set that established a negative association between ENSO events and rainfall in Bangladesh [49]. However, another study showed a positive correlation between SST in the Bay of Bengal with June rainfall in south-eastern Bangladesh, but no significant relationship with May, July, August and September rainfall [50]. Results have shown that the Indian summer monsoon and ENSO are negatively correlated [51]. Therefore, it is clear that this parameter exerts different effects in different places at different times.

The main climatic variables associated with malaria transmission on the Indian sub-continent are rainfall, temperature and humidity [22]. Studies on the interaction of climate and malaria in Bangladesh are limited because of a paucity of malaria case data, lack of skilled manpower and meteorological stations in endemic districts. In the Chittagong Hill Tracts, for example, there is only one meteorological station situated in Rangamati. However, the potential role of climate change and its impact on health, particularly malaria, has received increasing attention in Bangladesh.

It seems counterintuitive that a low NDVI, an indicator of low vegetation greenness, is associated with increases in malaria cases, since the primary vectors in Bangladesh, such as *An. dirus*, are associated with forests. However, NDVI is a reasonably reliable indicator of rainfall and the unadjusted analysis both indicate a relationship between rainfall and malaria cases: i.e. as rainfall (and NDVI) decreases malaria increases. This relationship can be explained by the drying up of rivers and streams creating suitable breeding sites for the vector fauna. Bangladesh has very high vector species diversity and vectors suited to these habitats may be responsible for the observed results.

The current study displays several limitations. First, the cases were based on one hospital's data. Although the hospital is the reference hospital for all 10 sub districts of Rangamati district and the cases are somewhat representative of the entire district, this sample may be underrepresented as only the severe cases are likely to be referred to the hospital. However, whether or how this would introduce a temporal trend in the data that would distort the results is not evident. Second, *P. vivax* and *P. falciparum* malaria cases were pooled together as total malaria cases. This may confound more detailed interactions of these two parasites, their vectors and climatic conditions. However, environmental conditions permitting parasite development broadly overlaps with *P. vivax* being somewhat more permissive in its temperature tolerances. Furthermore, as more than 90% of malaria cases are due to *P. falciparum* and the proportion of *P. vivax* is very small in this area, separate analysis may not be statistically meaningful. Third, the study was based on only one district, the Chittagong Hill Tracts, so that extrapolating these results to other parts of the country or south Asia needs to be done with caution. This region, however, includes areas with some of the highest rates of malaria transmission and so is of practical importance in itself. Fourth, there may be concerns about a possible effect of non-climatic factors such as land use changes, population growth, development of drug resistance, change in diagnostic criteria, changes in local health infrastructure, access to care and public health interventions over the duration of the 20 year study. However, as these factors are not likely to change on a monthly interval, and it seems unlikely that they would obscure the short-term dependence of malaria on the factors investigated in this study.

## Methods

### Malaria and Climate Data

All malaria cases were collected from the Rangamati district hospital (22° 40′ N, 92° 11′ E) from January 1989 to December 2008. This hospital is the reference hospital for all 10 sub-districts of Rangamati district. There is no other hospital or clinic in the central town of Rangamati. Not all cases were confirmed by microscopy. From 1988 to 2004, cases were characterized as uncomplicated malaria (UM), treatment failure malaria (TFM) and severe malaria (SM). Uncomplicated malaria was presumptively determined while TFM and SM were confirmed by malaria microscopy. From 2004 to 2009, cases were confirmed as uncomplicated malaria presumptive (UMP), uncomplicated malaria confirmed (UMC), as well as SM and VM (vivax malaria). Neither microscopy nor rapid diagnosis tests (RDT) were performed for UMP, but for the others either microscopy or RDT was used for diagnosis. A survey conducted by the authors in one of the thana (Rajathali sub-district) in Rangamati district showed that 93.2% of malaria cases were *P. falciparum*, 1.9% were *P. vivax* and 5.0% were mixed infections (unpublished data). Monthly climatic data including rainfall, temperature, and relative humidity were obtained from the Bangladesh Meteorological Department. The meteorological station is in the central town of the district within 5 km of the study hospital. Normalized difference vegetation index was derived from the data library of the International Research Institute (IRI) of Lamont Doherty Earth Observatory (LDEO) at Columbia University, USA. Mean monthly SSTs in the Bay of Bengal (20–21°N, 90–91°E) were derived from the NOAA Optimum Interpolation Sea Surface Temperature dataset [52], [53]. The strength of ENSO was measured using SST in the Niño 3 region (NIÑO3) in the Pacific Ocean, which were extracted from NOAA climate prediction center datasets [54].

### Statistical Analysis

The climatic data and malaria case time series data were computerized and cross checked. Seasonality and peaks of malaria cases as well as different climate parameters in the study period were observed graphically using scatter plots. Due to over-dispersed data for monthly malaria cases, a generalized linear negative binomial regression model was developed using the number of monthly malaria cases and climatic parameters. Potentially significant associations were analyzed by comparing patterns of variation in incidence of malaria over time with the patterns of each climatic parameter, using time series regression analysis. The unit of analysis in this study was the month, thus potential confounders that must be controlled are those that vary over time, possibly coinciding with each climate variable. Thus the association between the particular climatic parameter (e.g. temperature) and malaria incidence can be confounded by the other climatic parameters (e.g. rainfall, humidity and NDVI).

Temporal associations between climate and disease can be confounded by trends and seasonal patterns. To account for seasonality of malaria that was not directly linked with the climate, we included indicator variables for each month in the model. Indicator variables for the years of the study were also incorporated into the model to allow for long-term trends and other variations between years. To allow for autocorrelations, an autoregressive term at order 1 was incorporated into the model [55].

### Models for temperature, rainfall, humidity, NDVI and SST

From exploratory analyses, we considered lag times, (the delay in the effect of climate factors on the number of malaria cases) of up to 3 months for temperature, rainfall, humidity, NDVI and SST of the Bay of Bengal. In our initial analyses, we fitted a natural cubic spline (3 df) [56] to the average climatic factors over lag times of 0–3 months. Natural cubic splines were used to create graphs, where the number of malaria cases was plotted as smoothed functions of climatic factors [56], to visually assess the functional form of the adjusted relationship, thereby identifying whether the relationship was likely to be linear or not across the full range of independent variables. Finally, potential mutual confounding between temperature, rainfall, humidity and NDVI were adjusted to identify independent associations of monthly malaria cases and each particular climatic parameter. Since the SST of the Bay of Bengal was regarded as a more distant factor (compared with local climatic factors), we did not adjust it for the effect of the local climatic parameters of temperature, rainfall, humidity and NDVI. The final model was:
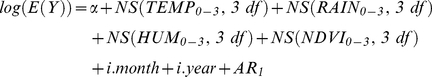
(1)

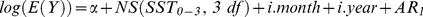
(2)
*E(Y)* is the expected monthly case count, NS indicates a natural cubic spline function, *TEMP_0–3_*, *RAIN_0–3_*, *HUM_0–3_*, *NDVI_0–3_* and SST*_0–3_* represent average temperature, rainfall, relative humidity, NDVI and SST at lag 0–3 months, respectively, *i.month* represents indicator variables for the month, *i.year* represents indicator variables for the year, and *AR_1_* represents a first-order autoregressive term. For example, *NS(TEMP_0–3_, 3 df)* indicates a linear term (raw data) and two spline terms of temperature at a lag of 0–3 months.

We then fitted the data to the linear threshold models i.e., models that assume a log-linear increase in risk. The increase in the number of malaria cases associated with 1% decrease in a given measure of climatic parameters (estimated as coefficients from the regression model) was reported as a percentage change.

### Model for NINO3

We considered lag times of up to 11 months for NINO3. We fitted a natural cubic spline (3 df) [56] to the average NINO3 over each 4-month period (i.e. lags 0–3, 4–7 and 8–11 months), as separate splines that were simultaneously included in the model.
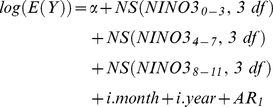
(3)To investigate whether the results were sensitive to the levels of control for seasonal patterns, analyses were repeated using Fourier terms of the month up to the fifth harmonic per year, adding one harmonic at a time. Diagnostics for model (1) including plots of model residuals, predicted and observed time series plots, partial autocorrelation function of the residuals were calculated (Figure S2). All analyses were performed by STATA 10 (Stata Corporation, College Station, Texas).

## Supporting Information

Figure S1 Time series of the average sea surface temperature (SST) of the Bay of Bengal and NINO3, 1989–2008.
(0.11 MB TIF)

Figure S2 Diagnostics of malaria-climate (temperature, rainfall, humidity and NDVI) models: (a) plots of model residuals, (b) predicted and observed time series plots, (c) partial autocorrelation function of the residuals.
(0.12 MB TIF)
