# Supplementary figures and images for: The Role of Climate Variability in the Spread of Malaria in Bangladeshi Highlands
Source: PLoS One. 2010 Dec 16;5(12):e14341. doi: 10.1371/journal.pone.0014341 (PMC3002939; doi:10.1371/journal.pone.0014341)

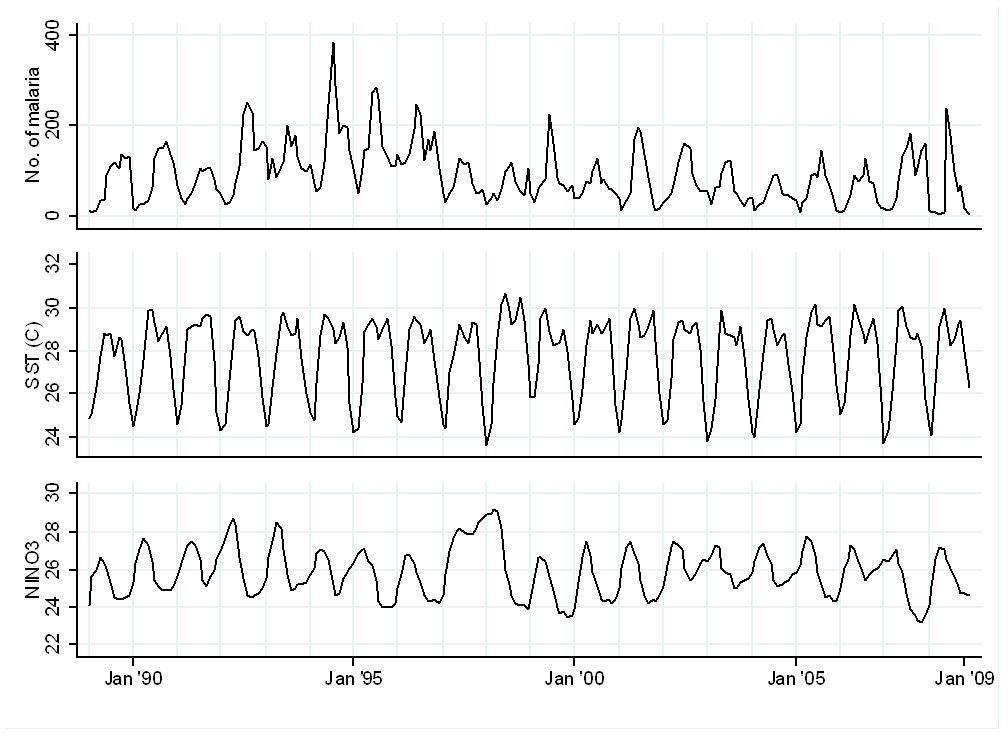

Supplement: Figure S1 — Time series of the average sea surface temperature (SST) of the Bay of Bengal and NINO3, 1989–2008. (0.11 MB TIF) [file pone.0014341.s001.tif]

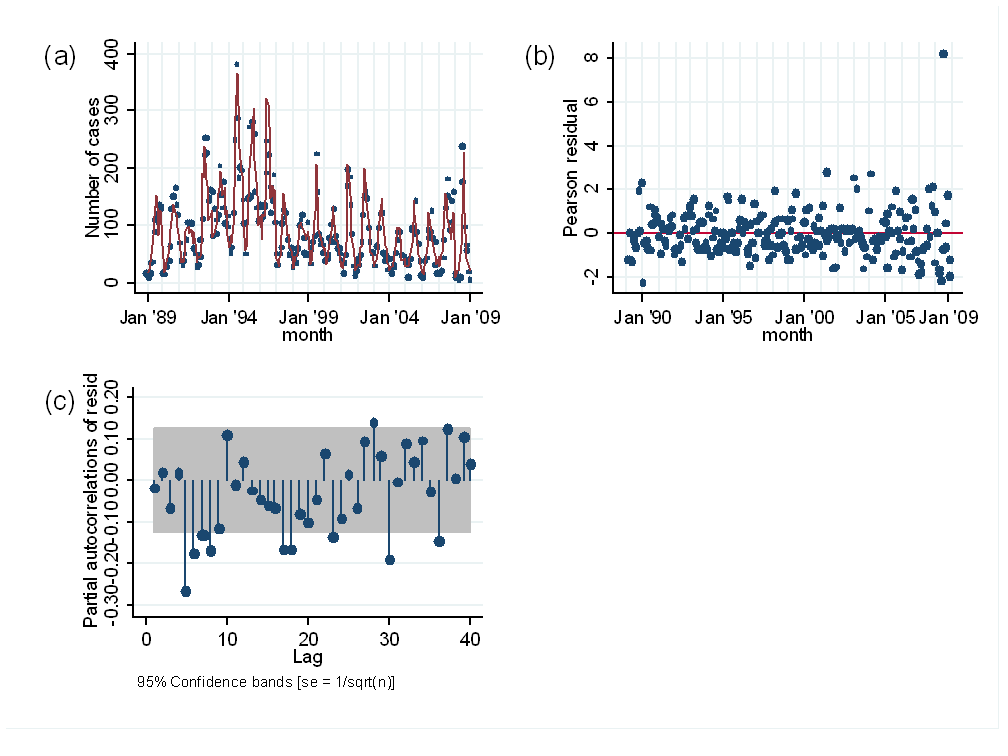

Supplement: Figure S2 — Diagnostics of malaria-climate (temperature, rainfall, humidity and NDVI) models: (a) plots of model residuals, (b) predicted and observed time series plots, (c) partial autocorrelation function of the residuals. (0.12 MB TIF) [file pone.0014341.s002.tif]
